# A Guide to Diseases of the Nose and Throat, and Their Treatment

**Published:** 1907-03

**Authors:** 


**A Guide to Diseases of the Nose and Throat, and their Treatment.** By Charles A. Parker, F.R.C.S. Edin. Pp. xiv., 624. Illustrated. Edwin Arnold. 1906.

A very practical exposition of diseases of the nose and throat, and their treatment by methods advocated at the Golden Square Throat Hospital, founded on lectures delivered at that hospital, has been written by a surgeon whose experience enables him to speak with authority, and this volume is sure to prove very valuable to all practitioners and students. It is clearly written, well illustrated, and well printed, although amongst the illustrations many will be recognised as old acquaintances, and some are distinctly poor, and not worthy of a modern text-book. Throughout the book the numerous formulæ for local pigments, sprays and applications will be found of service in practice. The very clear and detailed description of most of the technical methods now very frequently adopted by English laryngologists must prove helpful to those whose earlier studies of the speciality require to be kept abreast of the times, and hardly any page can be read by such without gleaning some point of value.

